# Vigilance decrement and mind-wandering in sustained attention tasks: Two sides of the same coin?

**DOI:** 10.3389/fnins.2023.1122406

**Published:** 2023-03-28

**Authors:** Víctor Martínez-Pérez, Almudena Andreu, Alejandro Sandoval-Lentisco, Miriam Tortajada, Lucía B. Palmero, Alejandro Castillo, Guillermo Campoy, Luis J. Fuentes

**Affiliations:** Facultad de Psicología, Universidad de Murcia, Murcia, Spain

**Keywords:** sustained attention to response task (SART), vigilance decrement, mind-wandering, alpha-band power, HD-tDCS

## Abstract

**Background:**

Decrements in performance and the propensity for increased mind-wandering (i.e., task-unrelated thoughts) across time-on-task are two pervasive phenomena observed when people perform vigilance tasks. In the present study, we asked whether processes that lead to vigilance decrement and processes that foster the propensity for mind-wandering (MW) can be dissociated or whether they share a common mechanism. In one experiment, we introduced two critical manipulations: increasing task demands and applying anodal high-definition transcranial direct current stimulation (HD-tDCS) to the left dorsolateral prefrontal cortex.

**Method:**

Seventy-eight participants were randomly assigned to one of four groups resulting from the factorial combination of task demand (low, high) and stimulation (anodal, sham). Participants completed the sustained attention to response task (SART), which included thought probes on intentional and unintentional MW. In addition, we investigated the crucial role of alpha oscillations in a novel approach. By assessing pre-post resting EEG, we explored whether participants’ variability in baseline alpha power predicted performance in MW and vigilance decrement related to tDCS or task demands, respectively, and whether such variability was a stable characteristic of participants.

**Results:**

Our results showed a double dissociation, such that task demands exclusively affected vigilance decrement, while anodal tDCS exclusively affected the rate of MW. Furthermore, the slope of the vigilance decrement function and MW rate (overall, intentional and unintentional) did not correlate. Critically, resting state alpha-band activity predicted tDCS-related gains in unintentional MW alone, but not in vigilance decrement, and remained stable after participants completed the task.

**Conclusion:**

These results show that when a sustained attention task involving executive vigilance, such as the SART, is designed to elicit both vigilance decrement effects and MW, the processes leading to vigilance decrement should be differentiated from those responsible for MW, a claim that is supported by the double dissociation observed here and the lack of correlation between the measures chosen to assess both phenomena. Furthermore, the results provide the first evidence of how individual differences in alpha power at baseline may be of crucial importance in predicting the effects of tDCS on MW propensity.

## Introduction

When people perform sustained attention tasks, two processes usually occur across time-on task: performance is affected by decrement in vigilance, and task-unrelated thoughts divert attention from the ongoing task, a process that is referred to as mind-wandering (hereafter, MW) (Smallwood and Schooler, 2015; Krimsky et al., 2017). These two phenomena are usually assessed through vigilance tasks, such as the sustained attention to response task (SART) (McVay and Kane, 2013; Lara et al., 2014; Axelrod et al., 2015; Seli et al., 2016; Jin et al., 2020; Filmer et al., 2021; Martínez-Pérez et al., 2021, 2022). In the SART, participants are asked to respond to the appearance of different numbers on the screen (go trials), except for digit 3, the target, in which case they should refrain from responding (no-go trials). MW is inferred by periodically asking participants whether they were focused on the task or had task-unrelated thoughts at the moment of the probe (Seli et al., 2016). The typical effect we find is an increase in the error rate, along with an increased report of MW over the course of the task. This means that as people’s vigilance decreases, their propensity for MW tends to increase (Krimsky et al., 2017). Behavioral and neuroimaging studies suggest that these two phenomena covary and overlap in their association with poorer task performance (Pattyn et al., 2008; Smallwood et al., 2008; Barron et al., 2011; McVay and Kane, 2012; Bogler et al., 2017). At the neural level, EEG alpha-band activity during the task has been described as a reliable electrophysiological correlate of both vigilance decrement (Schmidt et al., 2009; Craig et al., 2012; Correa et al., 2014; Wascher et al., 2014; Luna et al., 2020) and MW (Compton et al., 2019; Jin et al., 2019; Arnau et al., 2020; Groot et al., 2021), and poor performance on vigilance tasks has been associated with increased EEG alpha power. In addition, alpha oscillations have been causally related to increased vigilance when people are at low levels of arousal (Martínez-Pérez et al., 2022) and prevent impaired performance in sustained visual attention tasks (Clayton et al., 2019).

In this scenario, a relevant question is whether the processes leading to vigilance decrement and MW as a function of time-on-task share a common mechanism, i.e., both phenomena are “two sides of the same coin,” or on the contrary they can be dissociated, i.e., they can be considered as two distinct phenomena co-occurring with each other in the same task (Jin et al., 2019). A recent theoretical framework has addressed this question. Thomson et al. (2015) integrated a wide range of empirical findings on the decline of performance over time-on-task and explained them through their “resource control” model. This model argues that MW would be our default state and that, when dealing with vigilance tasks, we exert executive control to avoid this bias and keep the task goals in mind. Furthermore, these authors claim that the amount of resources available to perform a vigilance task is fixed and that the act of MW consumes those resources (Smallwood and Schooler, 2006). Therefore, across time-on-task, we devote fewer resources to task requirements and MW increases because our executive control decreases, and it is because of this conjunction of events that vigilance decreases. As we can surmise, this model proposes that MW and vigilance decrement are two highly dependent phenomena as they share a common mechanism that would allocate available resources. However, to date, empirical evidence on this theoretical model remains scarce (Luna et al., 2022b). In favor of this model are studies showing that MW increases as a function of time-on-task (Cunningham et al., 2000; McVay and Kane, 2012; Risko et al., 2012). For example, Thomson et al. (2014) found that in both a singleton search task and a flanker interference task (Eriksen and Eriksen, 1974), there was a higher proportion of MW and worse accuracy on final trials compared to that observed on initial trials. They found that MW strongly predicted changes in performance over time. Krimsky et al. (2017) also found an increase in MW and a decrease in accuracy over time in a working memory task. The authors stated that the results show empirical support for the resource control model by demonstrating a greater increase in MW in the more demanding condition, as it would lead to greater fluctuation in executive control. Martínez-Pérez et al. (2021) distinguished between intentional and unintentional MW (Seli et al., 2016) and assessed their time course in a vigilance task with executive demands [SART (Robertson et al., 1997)] and in another task involving arousal vigilance [psychomotor vigilance task, PVT (Lim and Dinges, 2008)]. They found a significant increase in the rate of unintentional MW (presumably stemming from a failure of executive control) toward the end of the SART (high executive demand), whereas the rate of unintentional MW remained stable in the low executive demand task (PVT). These authors argued that their results extend in a sense the support for Thomson’s resource control theory (Thomson et al., 2015) by including the intentionality aspect of MW.

On the other hand, other lines of evidence have claimed that vigilance decrement and MW are two phenomena that simply co-occur under certain conditions (Neigel et al., 2019). Interestingly, recent work has shown that sleepiness and MW, which often co-occur and are associated with poorer vigilance performance, are also independent predictors of performance on the SART, showing additive effects (Stawarczyk and D’Argembeau, 2016). A previous study found that background music decreased reports of MW but did not affect the effects of time-on-task (reaction times, RTs) when participants performed the PVT (Kiss and Linnell, 2021). Furthermore, the relationship between task demands, vigilance decrement and MW was studied in a recent machine-learning analysis of EEG research (Jin et al., 2020). The analysis showed that, although behavioral measures of MW, vigilance and task demands covaried, these factors were not associated with similar neural correlates.

The recent introduction of tDCS in MW modulation research has yielded some interesting findings (Kam et al., 2022). This non-invasive brain stimulation technique is believed to be capable of modulating neuronal network activity (Liu et al., 2018). Several studies provide evidence for the success of MW modulation by tDCS [see Axelrod et al. (2015), Kajimura and Nomura (2015), Kajimura et al. (2016, 2019), Bertossi et al. (2017), Filmer et al. (2019, 2021), Boayue et al. (2021), although see Boayue et al. (2020), Coulborn et al. (2020)]. As for the regions where tDCS stimulation has been applied, the most frequent are the left dorsolateral prefrontal cortex (l-DLPFC) (Axelrod et al., 2015; Filmer et al., 2019, 2021; Boayue et al., 2020) and the inferior parietal lobe (Kajimura and Nomura, 2015; Kajimura et al., 2016, 2019; Chou et al., 2020; Coulborn et al., 2020). When anodal tDCS was applied to the l-DLPFC, MW tended to increase, whereas when anodal tDCS was applied to the inferior parietal lobe, MW propensity decreased. Of particular interest, in this line of experiments on the modulation of MW propensity by tDCS, are the null effects of stimulation on performance in the vigilance task. Again, this pattern of results would partially support that processes that lead to vigilance decrement and those that foster the propensity to mind-wander should be differentiated.

Here, we wanted to investigate this discrepancy in the results of previous studies by employing an experimental design that would allow us to assess the relationship between vigilance decrement and MW in a single experiment. We used a combination of behavioral and neurophysiological measures in which we applied a high-definition tDCS (HD-tDCS) protocol to supposedly increase the propensity for MW and also manipulated task demands to affect decrement vigilance effects. We also recorded pre-post resting-state EEG data. Specifically, we administered anodal HD-tDCS over the l-DLPFC while participants performed one of two versions of the SART that differed in task demands. Periodically, we added thought probes to measure the propensity for MW. Since previous studies had observed qualitative differences between intentional and unintentional MW (Seli et al., 2016; Martínez-Pérez et al., 2021), we included thought probes to detect both types of MW. We also assessed participants’ working memory capacity in a first session, as recent studies have shown that the propensity for MW is related to working memory capacity and task demands (Ju and Lien, 2018; Robison and Unsworth, 2018; Soemer and Schiefele, 2020). We hypothesize that if the two manipulations described above (brain stimulation and task demand) affect both MW rate and vigilance decrement, our data would support the proposition that both phenomena share a common mechanism, which would be in line with the model of Thomson et al. Conversely, if each of our manipulations exclusively affects one of these factors and not the other, this double dissociation would run counter to the concept of a common mechanism underlying both the vigilance decrement function and the MW propensity.

A second aim of the present study was to examine the relationship between participants’ variability in alpha-band power baseline as a predictor of performance on the sustained attention tasks and tDCS-related gains in MW, as previous studies have revealed the relevance of differences in baseline in estimating gains from cognitive training in general (Colzato et al., 2020) and non-invasive brain stimulation in particular (Harty and Cohen Kadosh, 2019; Martínez-Pérez et al., 2022). We recorded resting-state EEG before and after participants completed the version of the SART to determine whether resting-state alpha-band activity is a stable personal trait of participants.

## Materials and methods

### Participants

Eighty healthy adults (selected between 18 and 30 years old), undergraduates at the University of Murcia, volunteered to participate in this study. Two of them did not complete the second session of the experiment, leaving a final sample of 78 participants (65 females) with an average age of 19.8 years. A *post hoc* sensitivity analysis was performed with G*Power 3.1.9.7 (Faul et al., 2007), with alpha = 0.05, and 1-β = 0.8, and showed that the minimum effect size that could be detected with the current sample (*N* = 78) was *f* = 0.32 (η^2^ = 0.093) for the critical stimulation (2) × task demand (2) interaction. All participants gave written informed consent and were compensated with course credits to participate in this study. All participants had normal or corrected-to-normal vision. They were also screened for any of the established exclusion criteria for HD-tDCS: pregnancy, epilepsy, medication, pacemaker use, and history of neurological or psychiatric disorders. The study was approved by the Ethics Committee of the University of Murcia and was conducted in accordance with the approved guidelines and the Declaration of Helsinki.

### Procedure

Participants attended the laboratory in two sessions, during which the approved protocol for the prevention of COVID-19 was followed. In the first session, to assess their working memory capacity, participants performed two complex span tasks: symmetry span and rotation span tasks (Kane et al., 2004). In the symmetry span task, participants were asked to recall the location of several sequentially lightened squares on a 4 × 4 grid, after which they performed a distraction task in which they had to decide whether or not a figure was symmetrical along a vertical axis. After two to five trials, participants were asked to reproduce the sequence of squares in the grid that they had previously memorized. In the rotation span task, similar to the symmetry span, participants first had to memorize the direction and length of several arrows, after which they performed a distraction task in which they decided whether a capital letter was normally rotated or mirrored. After responding to two to five letters (rotated or mirrored), participants had to recall the direction and length of previously presented arrows in the same order in which they had memorized them. Both memory tasks were obtained from Engle’s attention and working memory laboratory,^1^ and lasted approximately 12 min each, including instructions and a practice block followed by three experimental blocks.

**FIGURE 1:**
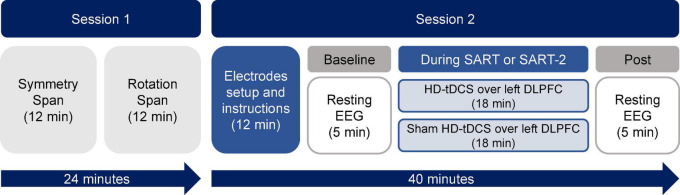
Schematic illustration of the experimental procedure.

The second session consisted of the application of HD-tDCS while participants performed one of two different versions of the SART with periodic thought probes (see the next section for details). Resting EEG signals were recorded pre- and post-brain stimulation. The procedure of the first and second sessions is shown schematically in Figure 1. Participants were randomly assigned to one of four groups formed from the factorial crossing of the factor stimulation (anodal or sham) and the factor task demand of the SART (high or low). The resulting groups were anodal-high, sham-high, anodal-low and sham-low. The experimental tasks were performed on a 22-inch TFT monitor with a resolution of 1,920 × 1,080 pixels at a viewing distance of approximately 60 cm. We programmed the tasks and analyzed the data using E-Prime-3. Responses were recorded with a 5-button Chronos device (Psychology Software Tools).

In the SART, we manipulated the proportion of go trials to obtain a low (7/9) or high (8/9) demanding version of the task. Wilson et al. (2016) showed a critical interval in the proportion of go trials between 0.8 and 0.95 in which errors increased dramatically and RTs decreased. Here, in the low-demand condition, the proportion of go trials was 0.77 (below the critical interval), and in the high-demand condition it was 0.88 (within the critical interval). In each version of the SART, the digits 1 to 9 were presented in the center of the screen for 250 ms. Each digit was presented 100 times for a total of 900 stimuli. The digits appeared randomly in different font sizes (18, 27, 36, 45, and 54 points; Consolas). After each digit, a mask (a circle with a diagonal cross in the center) was displayed for 800 ms, followed by the presentation of a blank screen for 100 ms. The stimuli were presented in the center of the screen in white on a black background. Participants were required to respond to each digit by pressing the right button on the response box (go trials) except when digit 3 (high-demand version) or digits 3 or 7 (low-demand version) were presented, in which case participants were required to refrain from responding (no-go trials). Participants were encouraged to give quick responses but to make as few mistakes as possible. At the beginning of the task, 18 practice trials were included following the same procedure. Participants’ MW was assessed using thought probes. Each probe appeared randomly in cycles of 46 trials (20 thought probes in total). The question “Which of the following responses best characterizes your state of mind just prior to the presentation of this display?” appeared on the screen with three response options: “On-task,” “Intentional MW,” and “Unintentional MW.” Before starting, they were given verbal and written instructions both for answering the question and for the task itself. For the thought probe questions, participants were told that being “on-task” meant that they were thinking about something related to the task (how hard it was, how boring it was, or the buttons they had to press). Participants were then provided with a definition of MW and briefly explained the difference between intentional and unintentional MW. They were told that intentional MW refers to moments when they voluntarily think about things unrelated to the task (e.g., the shopping list) and unintentional MW to moments when they involuntarily think about things unrelated to the task (e.g., when a past event comes to mind). Participants were explicitly told that there were no right or wrong answers to the questions, but they should be honest in their responses. They were instructed to use the three buttons on the left of the response box (buttons 1, 2, and 3) to choose between the three response alternatives: on-task, intentional MW, or unintentional MW. Completing the task took about 18 min. For the time course analysis of the data, we divided the SART into four blocks of 225 trials (900 in total) and two halves of 10 thought probes (20 in total).

### HD-tDCS/EEG protocols

**FIGURE 2:**
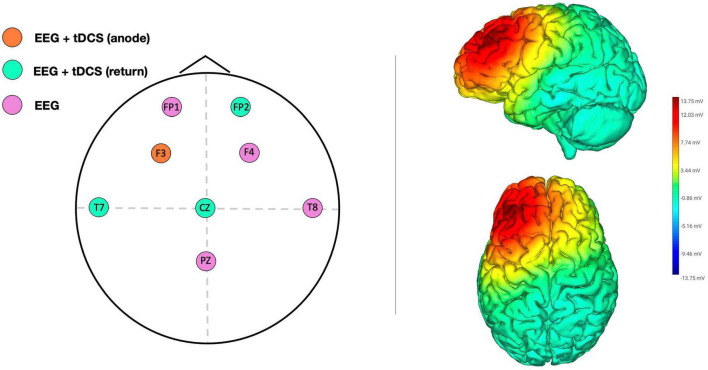
Electrode configuration for EEG recording and HD-tDCS montage according to the international EEG 10–20 system **(left panel)**. Simulation of the electric voltage field for HD-tDCS **(right panel)**.

High-definition transcranial direct current stimulation was administered *via* a Starstim^®^ wireless neurostimulator system (Neuroelectrics, Barcelona, Spain) connected to hybrid tCS/EEG NG Pistim circular electrodes (pi cm^2^). The target brain area for stimulation was the l-DLPFC, located in F3 according to the 10–20 system. Three return electrodes, T7, Cz, and Fp2, were placed in a triangular scheme, each with a 33% current return (Figure 2). Both the researcher and participants were unaware of the stimulation conditions (double blind design), although we did not check the efficacy of blinding by using a post-stimulation questionnaire to assess whether participants guessed the stimulation condition to which they had been assigned. The intensity was set at 1.5 mA, and anodal stimulation was applied online for 25 min from the SART onset (including 30 s up/down ramp). Sham stimulation was applied only during ramp periods to emulate the tingling sensation on the skin. This type of high-definition montage has been shown to improve the focality of stimulation (Kuo et al., 2013) and this particular montage was effective in modulating DLPFC in previous studies conducted in our laboratory (Martínez-Pérez et al., 2019, 2022).

EEG signals were recorded using the same Starstim^®^ 8-channel system (Neuroelectrics, Barcelona, Spain). In addition to the location of the stimulation (F3) and return electrodes (T7, Cz, and Fp2), additional EEG electrodes were placed at sites FP1, F4, T8, and Pz. The reference electrode was placed on the right earlobe using an EarClip electrode. The signals were recorded with a sampling frequency of 500 Hz, a bandwidth of 0–125 Hz, and a notch filter at 50 Hz. All electrode impedances were kept below 5 kΩ. Five minutes of resting-state EEG activity was recorded before and after stimulation for each participant (see Figure 1). Participants were instructed to remain still and keep their eyes open while looking at a fixation point located in the center of the screen.

Preprocessing of each record was conducted using the EEGLAB v2021.1 toolbox (Delorme and Makeig, 2004) in MATLAB. The dataset was imported to EEGLAB using the NE EEGLAB NIC plugin. Four participants had corrupted data in the recording and were removed from the analysis. We then re-referenced to an average of all the electrodes and applied a high pass filter at 1 Hz and a low pass filter at 30 Hz. Afterward, the filtered EEG signal was decomposed using independent component analysis (ICA) and reconstructed excluding components containing eye blinks.

Power spectral density (PSD) was calculated using the spectopo function implemented in the EEGLAB toolbox, which is based on Welch’s method. To determine whether tDCS and/or task performance had any effect on alpha power compared to baseline (i.e., the eight-electrode mean of resting-state alpha band power before stimulation), the normalized percentage change (see Equation 1) between resting-state signals in the alpha band (8–13 Hz) after stimulation compared with those registered at rest before stimulation was calculated.


(1) $$P\,e\,r\,c\,e\,n\,t\,c\,h\,a\,n\,g\,e=\frac{\left(P\,o\,w\,e\,r_{p\,o\,s\,t}-P\,o\,w\,e\,r_{p\,r\,e}\right)}{\left|P\,o\,w\,e\,r_{p\,r\,e}\right|}*100$$

In this way, we ensured that the differences between the groups were not determined by individuals with high baseline power (Clayton et al., 2019).

### Statistical analyses

Data were analyzed with JASP 0.16 adopting a significance level of α = 0.05. The main interest of the present study was to determine whether participants modulated their vigilance decrement and MW rate as a function of task demands and tDCS stimulation.

Two analyses of variance (ANOVAs) were conducted, one with accuracy on the no-go trials in the SART as the dependent variable and the other with MW rate as the dependent variable. In the first analysis, task demand (low, high) and stimulation (anodal, sham) were the between-participant factors, and trial block (1, 2, 3, and 4) was the within-participant factor. In the second analysis, task demand (low, high) and stimulation (anodal, sham) were again the between-participant factors, and half (first half, second half) and intentionality in MW (intentional, unintentional) were the within-participant factors. To control for possible effects of individual differences in working memory capacity, we introduced this measure as a covariate. Because working memory capacity did not produce any significant effect (*F*s < 1), we did not take this variable into account any further.

To examine whether individual differences in baseline alpha power predicted tDCS effects on MW propensity and vigilance decrement, regression analyses were conducted with baseline alpha power as a predictor and the slope of the vigilance decrement function or MW rate as dependent variables. The slope was the beta of the general linear model fitting the mean accuracy of each of the four blocks in the SART for each participant.

## Results

### Vigilance decrement

**FIGURE 3:**
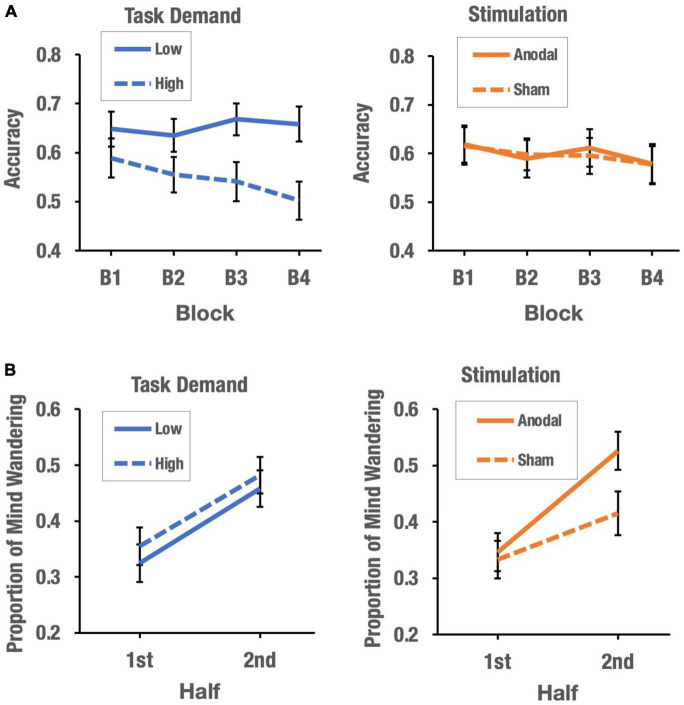
The effects of task demand and stimulation on vigilance decrement **(A)** and mind-wandering **(B)**.

The main effect of task demand was significant, *F*(1, 74) = 4.63, *p* = 0.035, η^2^ = 0.005. Participants showed greater accuracy in inhibiting no-go responses in the SART when task demands were low (*M* = 0.65) than when task demands were high (*M* = 0.55). More importantly, the effect of block × task demand was also significant *F*(3, 222) = 4.66, *p* = 0.004, η^2^ = 0.007. The analysis of the interaction showed that at high task demands, performance on the vigilance task decreased across blocks of trials, *F*(3, 111) = 5.45, *p* = 0.004, η^2^ = 0.123 (*M_*B1*_* = 0.59, *SD*_*B1*_ = 0.26; *M_*B2*_* = 0.55, *SD*_*B2*_ = 0.23*, M_*B3*_* = 0.54, *SD_*B3*_* = 0.25 and *M_*B4*_* = 0.50, *SD_*B4*_* = 0.25). Polynomial contrast showed that such a decrease in performance could only be explained by a significant linear component, *t*(117) = 3.98, *p* < 0.001. In contrast, performance on the vigilance task across blocks remained stable when task demands were low, which was supported by a non-significant effect of the block factor, *F*(3, 111) = 1.2, *p* = 0.31, η^2^ = 0.031 (*M_*B1*_* = 0.65, *SD_*B1*_* = 0.22; *M_*B2*_* = 0.64, *SD_*B2*_* = 0.21*; M_*B3*_* = 0.67, *SD_*B3*_* = 0.20*; M*_*B4*_ = 0.66, *SD_*B4*_* = 0.22). Neither the main effect of stimulation nor the interactions involving stimulation (stimulation × block, stimulation × task demand, and stimulation × task demand × block) reached statistical significance. Figure 3A shows the effects of task demand and stimulation on the vigilance decrement function in the SART.

### Mind-wandering

In a first analysis we introduced the type of MW (intentional, unintentional) as a factor in the analysis of variance (ANOVA). The rate of unintentional MW was significantly higher than the rate of intentional MW, *F*(1, 74) = 138.02, *p* < 0.001, η^2^ = 0.368. However, this factor did not interact with any other factor. Therefore, the main statistical analysis was performed on the overall rate of MW (intentional MW rate plus unintentional MW rate) as the dependent variable.

The main effect of half was significant, *F*(1, 74) = 33.72, *p* = 0.008, η^2^ = 0.005. Participants’ MW rate was higher in the second half of the task (*M* = 0.47) than in the first half (*M* = 0.34). Importantly, the stimulation × half interaction was significant, *F*(1, 74) = 4.66, *p* = 0.004, η^2^ = 0.007. The interaction analysis showed no significant differences between stimulation conditions in the first half of the task, *t*(76) = 0.27, *p* = 0.79, Cohen’s d = 0.061, but showed significant differences between anodal and sham conditions in the second half of the task, *t*(76) = 2.13, *p* = 0.03, Cohen’s d = 0.48. For both sham and anodal stimulation, the MW rate increased from the first to the second half of the task [sham: *t*(76) = 2.62, *p* = 0.011, M_first–half_ = 0.33, *SD* = 0.21; M_second–half_ = 0.42, *SD* = 0.21; anodal: *t*(76) = 5.73, *p* < 0.001 (M_first–half_ = 0.35, *SD* = 0.21; M_second–half_ = 0.53, *SD* = 0.24)], but the increase was greater in the anodal stimulation condition than in the sham condition. Neither the main effect of task demand nor the task demand × half, task demand × half × stimulation and stimulation × task demand interactions reached statistical significance. Figure 3B shows the effects of task demand and stimulation on the propensity for MW.

### Correlation between vigilance decrement and mind-wandering

To assess whether the two phenomena were correlated, we conducted separate correlation analyses between the slope of the vigilance decrement function and the rates of overall MW, intentional MW and unintentional MW. None of the correlations reached statistical significance (*r*_overallMW_ = −0.21, *p* = 0.064; *r*_intentionalMW_ = −0.16, *p* = 0.159; *r*_unintentionalMW_ = 0.033, *p* = 0.771).

### EEG data

First, we assessed whether anodal HD-tDCS had any aftereffect on alpha power. To test this hypothesis, a 2 (task demand: low, high) × 2 (tDCS stimulation: anodal, sham) between-participants ANOVA was performed with the pre-post percentage change in the alpha band as the dependent variable. Neither the main effects of stimulation [*F*(1, 70) = 1.65, *p* = 0.204, η^2^ = 0.022], task demand [*F*(1, 70) = 0.323, *p* = 0.572, η^2^ = 0.004] nor the stimulation × half interaction [*F*(1, 70) = 1.24, *p* = 0.270, η^2^ = 0.017] reached statistical significance. In addition, we calculated resting-state post-stimulation alpha power minus pre-stimulation alpha power in each condition. None of the differences were significantly greater than 0 (all *p*s > 0.05).

**FIGURE 4:**
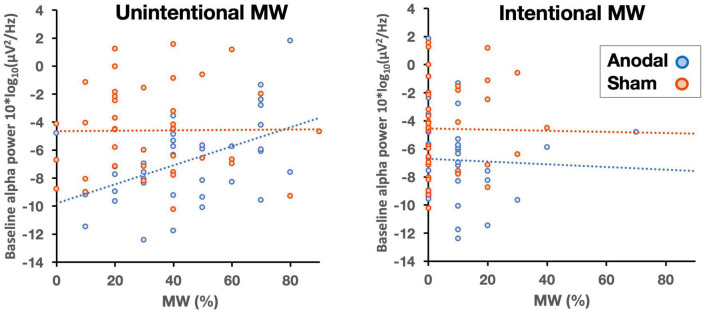
Correlations between baseline alpha power and unintentional **(left panel)** and intentional **(right panel)** MW (in percentages) as a function of stimulation (anodal, sham).

Next, we assessed whether the effects of stimulation on MW could be predicted by participants’ differences in alpha power at baseline (pre-stimulation recording). Because the effects of anodal HD-tDCS on MW were observed in the second half of the tasks, we performed a regression analysis with alpha power at baseline as the predictor and the proportion of MW in the second half of the tasks as the dependent variable. No effect was observed for the sham group (*F* < 1), but, more importantly, we found a significant effect in the anodal group *F*(1, 34) = 8.19, *p* = 0.007, *R*^2^ = 0.194. In addition, we examined the MW intentionality factor in this group of participants. We performed two additional regression analyses with intentional and unintentional MW rates in the second half of the tasks as dependent variables. Only the rate of unintentional MW was predicted by alpha power at baseline *F*(1, 34) = 10.05, *p* = 0.003, *R*^2^ = 0.228. This suggests that the increment in the rate of unintentional MW is due to an increase in alpha power and that this increment appears to be modulated by participants’ alpha power at baseline (Figure 4).

Finally, we assessed whether a similar relationship was also observed between the baseline alpha power and vigilance decrement using a regression analysis in which the slope of the vigilance decrement function was calculated across the four blocks of trials for each participant. Baseline alpha power was entered as the predictor, and the slope of the function was entered as the dependent variable. Importantly, the results showed that baseline alpha power did not predict the slope of the vigilance decrement function (*F* < 1). Thus, consistent with the behavioral results, the EEG results also point to a dissociation between vigilance decrement and MW.

## Discussion

Two main objectives were addressed in the present study. First, we asked whether distinct processes lead to vigilance decrement and to propensity for MW, two phenomena that may co-occur under the same experimental conditions, or whether both phenomena share a common mechanism. The second aim was to explore the role of resting-state alpha band activity in the interaction between vigilance decrement and MW in a sustained attention task such as the SART.

To address the first objective, we used two versions of the SART that varied in task demands and applied anodal or sham HD-tDCS stimulation to participants in a double-blind design. Thought probes were interleaved while participants performed the vigilance task to assess both intentional and unintentional MW. Our main finding showed that anodal tDCS stimulation on the l-DLPFC exclusively affected the propensity for MW, a result that replicates previous studies (Axelrod et al., 2015, 2018; Filmer et al., 2019, 2021). In turn, when we manipulated task demands, only vigilance decrement was affected, with a more abrupt decrease in performance occurring only in the high-demand version of the task (Caggiano and Parasuraman, 2004; Helton and Russell, 2011, 2013; Head and Helton, 2014; Luna et al., 2022a). Importantly, we controlled the experiment so that our results were not affected by individual differences in participants’ working memory capacity. In comparison to the aforementioned studies, which used a dual-task procedure with a concurrent working memory task, in the present study we manipulated task demands in such a way that it was as non-disruptive as possible to the dynamics of the SART and the periodic introduction of thought probes. Our results are similar to those found by Wilson et al. (2016); that is, performance across time-on-task was dramatically reduced when task demands were increased by manipulating the proportion of go trials. As these authors found, the rate of MW did not differ significantly when the proportion of go trials increased.

Taken together, these results do not support the hypothesis that a common mechanism underlies vigilance decrement and the propensity for MW. Furthermore, the results are generally consistent with previous studies showing that the effects of time-on-task on performance and MW are dissociable phenomena (Stawarczyk and D’Argembeau, 2016; Wilson et al., 2016; Neigel et al., 2019; Jin et al., 2020; Kiss and Linnell, 2021). From a theoretical standpoint, the results of the current experiment are not consistent with the resource control model (Thomson et al., 2015). Note that recent work evaluating this theory has focused on how executive control declined over the course of the task and has neglected the MW aspect (Luna et al., 2022b). Here, we have observed that vigilance decrement is an unreliable predictor of MW, a claim that is supported by the lack of a significant correlation between the measures used to assess both phenomena. Our results suggest that vigilance decrement is best explained in terms of attentional resources [see Neigel et al. (2019)]. Indeed, our results are consistent with the predictions of the resource-depletion model (Fortenbaugh et al., 2017; Esterman and Rothlein, 2019), i.e., given that vigilance tasks involve effort, conditions in which the task is more demanding deplete resources more rapidly and thus produce a more prominent vigilance decrement (Caggiano and Parasuraman, 2004). Consequently, we observed that when task demands increased, the decrease in vigilance was greater. Our results also challenge the view that because vigilance tasks are monotonous and boring, increasing task demands will reduce MW intrusions (Smallwood, 2010; Thomson et al., 2015). In our study, we observed that MW rates increased consistently across time-on-task, and no differences were found between low- and high-demand tasks. In contrast, our results suggest that MW is not an attentional resource-dependent phenomenon, but rather reflects a failure of the executive control system to adequately deal with task-unrelated thoughts (McVay and Kane, 2010).

Furthermore, by applying anodal HD-tDCS on the l-DLPFC, in combination with resting-state EEG recordings, we were able to better characterize and distinguish these two phenomena also at the neuronal level. Thus, our second aim was to address the relationship between vigilance decrement and MW propensity with alpha band oscillations. Previous studies related alpha band power to drops in vigilance (Schmidt et al., 2009; Craig et al., 2012; Correa et al., 2014; Wascher et al., 2014; Luna et al., 2018; Clayton et al., 2019) or MW (Compton et al., 2019; Jin et al., 2019; Arnau et al., 2020; Groot et al., 2021) while assessing online EEG task-related activity [although see Lim et al. (2013)]. In contrast, we aimed to assess resting-state alpha band activity with a pre- post-stimulation design. First, we examined how interindividual variability in alpha power at baseline may influence the effects of tDCS and task demands on vigilance decrement and the propensity for MW. Crucially, our results indicated that individuals’ baseline alpha power predicts increased MW due to anodal tDCS. Furthermore, participants who showed higher alpha power at baseline were those who increased their MW the most due to anodal HD-tDCS stimulation. It should be noted that this prediction was found exclusively in unintentional MW, which makes the distinction between intentional and unintentional a key aspect when studying MW (Seli et al., 2016, 2018; Martínez-Pérez et al., 2021).

These findings bring a new perspective to the debate on the replicability issue reported recently (Boayue et al., 2020). Overall, our study highlights for the first time three issues crucial to understanding the effects of tDCS over the l-DLPFC on MW propensity: time-on-task, MW intentionality, and alpha power at baseline. Mechanistically, a tentative explanation for this pattern of results is as follows. MW has been conceived as a state that occurs when internally triggered task-unrelated thoughts, generated by the default mode network, become available to consciousness (Smallwood, 2010). There is an increased likelihood of MW as task performance progresses. We speculate that, at some point, internal thoughts reach a threshold, and then the pathway by which task-unrelated thoughts enter consciousness is prioritized. These latent thoughts can find external facilitators to cross the threshold, as is the case when applying anodal tDCS on the l-DLPFC. Note that the DLPFC is a key structure of the executive control network (Miller and Cohen, 2001), and it has previously been suggested that MW may reflect a failure of the executive control system to cope with task-unrelated thoughts (McVay and Kane, 2010). Our results show that most of these thoughts appear spontaneously (unintentionally), although they may also arise deliberately (intentionally). Crucially, we have found a physiological marker, alpha power in the resting state, that predicts increases in unintentional MW when the MW rate is boosted by applying anodal tDCS stimulation. Therefore, we assume that the higher the baseline alpha power, the greater the likelihood that these spontaneous thoughts will exceed the threshold after brain stimulation. Why does alpha power work only in unintentional MW? The answer to this question might shed some light on the complex role of alpha oscillations in attention (Klimesch, 2012; Clayton et al., 2015, 2019). Given the spontaneous nature of unintentional MW, we speculate that alpha power at rest could be a marker of bottom-up processes related to attention. Furthermore, our results suggest that resting-state alpha power may indicate a stable natural disposition of individuals, i.e., a trait. In this regard, we found no significant differences in pre-post change in alpha power as assessed by normalized percentage change as a consequence of anodal stimulation or task demands. Taken together, our results are in line with a previous study that showed that higher levels of resting-state alpha power were associated with greater magnitudes of attentional blink (MacLean et al., 2012). It is important to note that the absence of pre-post changes in alpha power observed in the present study does not challenge previous findings that have observed an increase in alpha power across time-on-task (Compton et al., 2019; Jin et al., 2019; Arnau et al., 2020; Luna et al., 2020; Groot et al., 2021). It is possible that the increase in alpha power observed in those studies is because it was monitored online (Compton et al., 2019; Jin et al., 2019; Arnau et al., 2020; Luna et al., 2020; Groot et al., 2021). However, such an increase may fade quickly over time, once the task has ended, so that it is not maintained in offline after-effects. A similar pattern has been found in pupillometry experiments (Martin et al., 2022). Nonetheless, we cannot rule out that the lack of increase in alpha power with time-on-task can be partially explained by the lack of electrodes in the parietal and occipital regions. Recent studies have observed greater increases in parietal compared to frontal alpha power with time-on-task (Compton et al., 2019; Hemmerich et al., 2023).

Finally, it should be noted that baseline alpha power measures did not show a consistent relationship with SART performance. This finding further suggests that vigilance decrement and MW could be epiphenomenal and questions the previously found relationship between alpha power and vigilance decrement. In our view, some of the observed changes in alpha power across time-on-task could, in fact, be driven by the occurrence of MW, something that had not been elucidated previously due to the lack of reports *via* thought probes. Another interesting observation was made in the EEG-based machine learning study by Jin et al. (2019). These authors set out to classify two different mental states, on-task and MW, and found that alpha power was the EEG marker most predictive of a MW state. Based on the present results, we suggest that future studies aiming to study the relationship between alpha band oscillations and vigilant attention also take into account the occurrence of MW.

## Conclusion

This experiment was intended to delve deeper into the relationships between vigilance decrement and the propensity for MW. In light of the above evidence, these two phenomena do not seem to be as dependent as previously assumed. On the contrary, they appear to be two types of epiphenomena. Although the role of alpha oscillations in vigilant attention remains ambiguous, our data showed, to the best of our knowledge for the first time, that individual differences in the resting-state alpha band can influence the propensity for MW when anodal HD-tDCS was applied over the l-DLPFC. More generally, our results shed new light on the potential applications of non-invasive brain stimulation, highlighting the importance of accounting for individual differences at baseline to predict its effects on cognition.

## Data availability statement

The raw data supporting the conclusions of this article are available at https://osf.io/j4q9n/.

## Ethics statement

The studies involving human participants were reviewed and approved by the Ethics Committee of the University of Murcia. The patients/participants provided their written informed consent to participate in this study.

## Author contributions

VM-P: conceptualization, methodology, investigation, software, formal analysis, data curation, writing—original draft, and visualization. AA: conceptualization, methodology, and investigation. AS-L and MT: methodology, investigation, software, formal analysis, data curation, writing—original draft, and visualization. AC: software and formal analysis. LP: investigation and methodology. GC: conceptualization, methodology, and supervision. LF: conceptualization, writing—original draft preparation, writing—reviewing and editing, supervision, visualization, and funding acquisition. All authors contributed to the article and approved the submitted version.

## Footnotes

1
https://englelab.gatech.edu/taskdownloads
